# Structural characterization of core-bradavidin in complex with biotin

**DOI:** 10.1371/journal.pone.0176086

**Published:** 2017-04-20

**Authors:** Nitin Agrawal, Juha A. E. Määttä, Markku S. Kulomaa, Vesa P. Hytönen, Mark S. Johnson, Tomi T. Airenne

**Affiliations:** 1 Structural Bioinformatics Laboratory, Biochemistry, Faculty of Science and Engineering, Åbo Akademi University, Turku, Finland; 2 Faculty of Medicine and Life Sciences and BioMediTech, University of Tampere, Tampere, Finland; 3 Fimlab Laboratories, Tampere, Finland; Russian Academy of Medical Sciences, RUSSIAN FEDERATION

## Abstract

Bradavidin is a tetrameric biotin-binding protein similar to chicken avidin and bacterial streptavidin, and was originally cloned from the nitrogen-fixing bacteria *Bradyrhizobium diazoefficiens*. We have previously reported the crystal structure of the full-length, wild-type (wt) bradavidin with 138 amino acids, where the C-terminal residues Gly129-Lys138 (“Brad-tag”) act as an intrinsic ligand (*i*.*e*. Gly129-Lys138 bind into the biotin-binding site of an adjacent subunit within the same tetramer) and has potential as an affinity tag for biotechnological purposes. Here, the X-ray structure of core-bradavidin lacking the C-terminal residues Gly114-Lys138, and hence missing the Brad-tag, was crystallized in complex with biotin at 1.60 Å resolution [PDB:4BBO]. We also report a homology model of rhodavidin, an avidin-like protein from *Rhodopseudomonas palustris*, and of an avidin-like protein from *Bradyrhizobium sp*. Ai1a-2, both of which have the Brad-tag sequence at their C-terminus. Moreover, core-bradavidin V1, an engineered variant of the original core-bradavidin, was also expressed at high levels in *E*. *coli*, as well as a double mutant (Cys39Ala and Cys69Ala) of core-bradavidin (CC mutant). Our data help us to further engineer the core-bradavidin–Brad-tag pair for biotechnological assays and chemical biology applications, and provide deeper insight into the biotin-binding mode of bradavidin.

## Introduction

Avidins (Avds) are proteins produced in oviducts of birds, reptiles and amphibians, and in several different bacteria [1,2]. In nature, Avds are most stable in their tetrameric [1,3–5] and dimeric [6] forms. They have a high affinity for D-biotin (K_d_ = ~10^−15^ M for chicken Avd) [1,4,7], which makes them attractive proteins for numerous biotechnological applications [7–10]. The best studied Avds to date are the eukaryotic chicken Avd [1–3,11] and the bacterial streptavidin from *Streptomyces avidinii* [5,12,13]. As a secreted protein, chicken Avd is post-translationally modified by cleavage of the 24 amino acid N-terminal signal peptide and by glycosylation at Asn17; the resulting mature protein has 128 amino acids [1,14]. The 159 amino acid gene product of the full-length streptavidin, in turn, is naturally trimmed at both the N and C termini: the most studied form has 127 amino acids, only containing residues 13–139 of the full-length, and is referred to as core-streptavidin [5]. This naturally occurring truncated form of streptavidin has been shown to have low aggregate formation and high solubility, while retaining high affinity for biotin [15]. Moreover, the crystal structure of full-length streptavidin revealed that the 20-residue C-terminal extension (residues 139–159) binds on the surface of the protein and that residues 150–153 occupy the ligand-binding site of the same subunit—acting as an intrasubunit intrinsic ligand [16].

In addition to chicken Avd and streptavidin, other Avds have been characterized. They include the natural eukaryotic Avds from, for example, zebrafish (*Danio rario*) [17], frog (*Xenopus tropicalis*) [18], and mushroom (*Pleurotus cornucopiae*) [19], and the bacterial Avds, such as the dimeric rhizavidin (*Rhizobium etli*) [6], shwanavidin (*Shewanella denitrificans*) [20] and hoefavidin (*Hoeflea phototrophica*) [21]; the thermostable tetrameric burkavidin (*Burkholderia pseudomallei*) [22]; as well as bradavidin II (*Bradyrhizobium diazoefficiens*), which has a highly dynamic oligomeric structure [23]. Apart from the naturally occurring Avds, a number of genetically engineered Avds [7] have been produced, too. These include the dual-chain Avd (dcAvd) [24] and single-chain Avd (scAvd) [25], respectively with two and four simultaneously modifiable ligand-binding sites, the monomeric streptavidin [26–28], the steroid-binding Avd (sbAvd) [29,30] and an extremely thermostable and protease resistant chimeric Avd [31].

Bradavidin is a tetrameric Avd from a nitrogen-fixing bacterium (*B*. *diazoefficiens*) found in root nodules of soy beans [32]. Wild-type (wt) bradavidin, after cleavage of the 25-residue signal peptide, has 138 amino acid residues, of which the last ten C-terminal residues are known as the Brad-tag [33]. The Brad-tag (^129^GSEKLSNTKK) binds to the ligand-binding site of a neighboring subunit and hence serves as an intrinsic, *intersubunit* ligand, dissimilar both in mode of interaction and sequence to the C-terminal sequence found in full-length streptavidin [16] that acts as an intrinsic *intrasubunit* ligand. The key residues of Brad-tag interacting with the ligand-binding site are Glu131, Lys132 and Leu133, whereas in full-length streptavidin the key residues are Asn150, Gly151, Asn152, and Pro153. In the case of streptavidin, several peptide tags have been developed, including strep-tag I [34], strep-tag II [34,35], Nano-tag [36] and SBP-tag [37]; all of which have a different binding mode in comparison to the Brad-tag.

Here, we report the tetrameric X-ray structure of core-bradavidin in complex with biotin at 1.60 Å resolution [PDB:4BBO]. In comparison to the X-ray structure of wt bradavidin (tetramer; 138 amino acids/14 kDa per subunit), core-bradavidin is artificially truncated at the C-terminus containing only residues 1–118 (12 kDa per subunit) and hence missing the Brad-tag [32]. In addition to these bradavidin structures, three structures of bradavidin II (each 115 amino acids/13 kDa per subunit) are known: two different crystalline forms of the apo protein, the monomeric Form-A [PDB:4GGR] and the dimeric Form-B [PDB:4GGT], as well as a tetrameric structure in complex with biotin [PDB:4GGZ] [23]. All the known X-ray structures of bradavidin and bradavidin II are from the same bacterium (*B*. *diazoefficiens* sp. nov; this strain was earlier known as the strain USDA 110 of *Bradyrhizobium japonicum* [38]). The core-bradavidin structure not only gives insight into the detailed biotin-binding mode of bradavidin but also helps us to further engineer core-bradavidin as a receptor with tighter binding towards ligands such as the Brad-tag itself. We also report homology models for the avidin-like proteins from *Rhodopseudomonas palustris* (rhodavidin [39]) and *Bradyrhizobium sp*. Ai1a-2 (referred to here as bradavidin A2); both of these proteins have the Brad-tag sequence at their C-terminus. Our better understanding about bradavidin binding to different ligands may aid in the development of novel constructs providing additional, improved tools for biotechnological purposes.

## Results

### Overall structure of core-bradavidin

**Fig 1:**
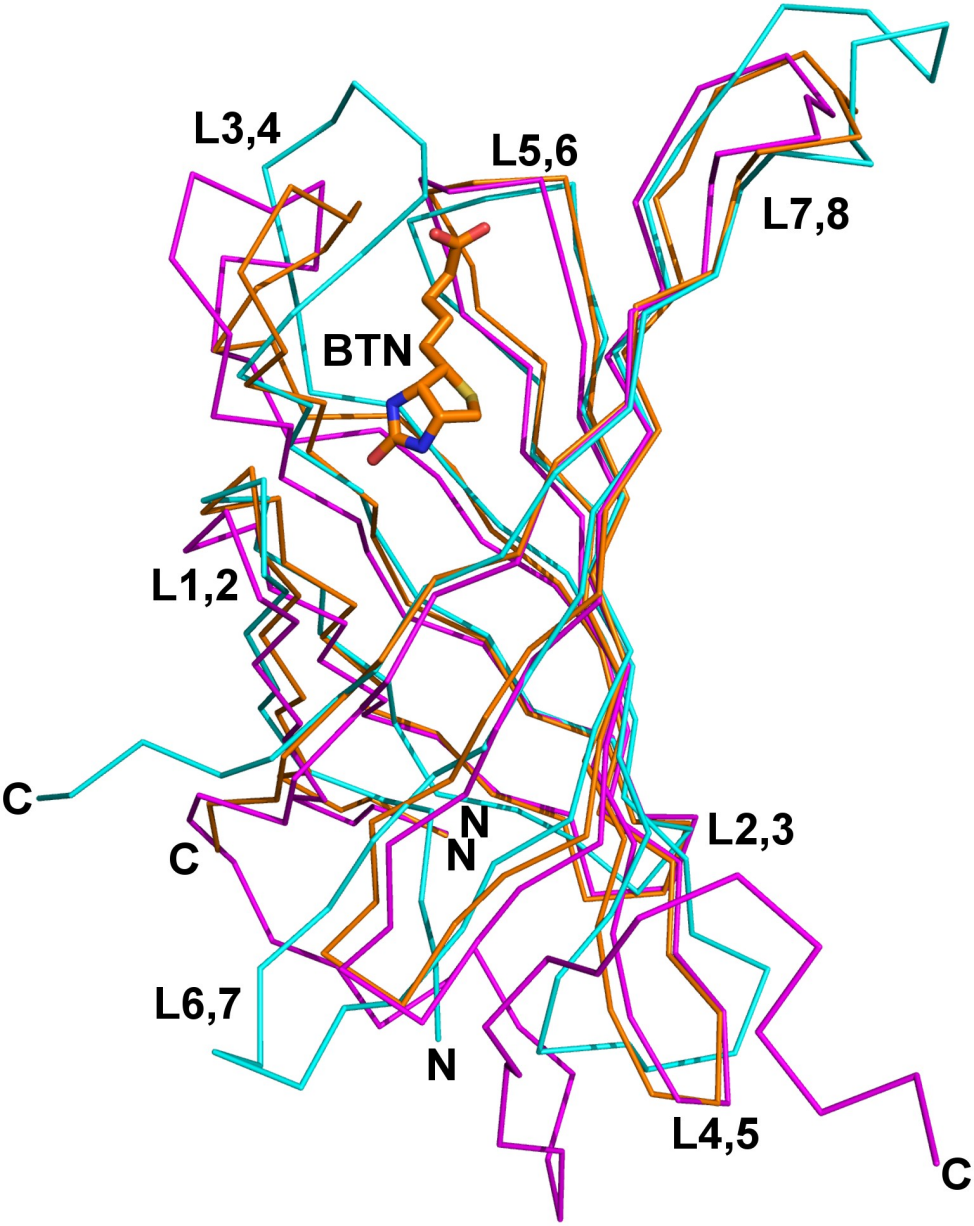
Superimposition of the Cα traces of subunit I of core-bradavidin (orange) [PDB:4BBO], wt bradavidin (magenta) [PDB:2Y32] and chicken Avd (cyan) [PDB:1AVD]. For clarity, only the biotin bound to core-bradavidin is shown (stick model). The loop regions L1,2 to L7,8, and the N and C termini, are labelled.

The 3D structure of core-bradavidin in complex with biotin was solved at 1.60 Å resolution. This structure represents an artificially truncated form of bradavidin and lacks the C-terminal residues Gly114-Lys138 (residues Gly129-Lys138 correspond to Brad-tag) [32]. The sequence of the solved structure shares 35% identity with chicken Avd [PDB:1AVD] [3], and the overall tetrameric structure and the folds of the individual subunits I-IV (numbering according to [4]) of core-bradavidin are typical for Avds, including the wt bradavidin structure reported in [33] (Fig 1). We have also tried to crystallize wt bradavidin in complex with biotin without success, which may have been due to the presence of the biotin-competing C-terminal Brad-tag sequence in the wt bradavidin. It is also possible that reconfiguration of the C-terminus of wt bradavidin occurred due to biotin binding and that this may have altered crystal contacts and affected crystal formation.

### Biotin-binding mode of core-bradavidin—Conserved features

**Fig 2:**
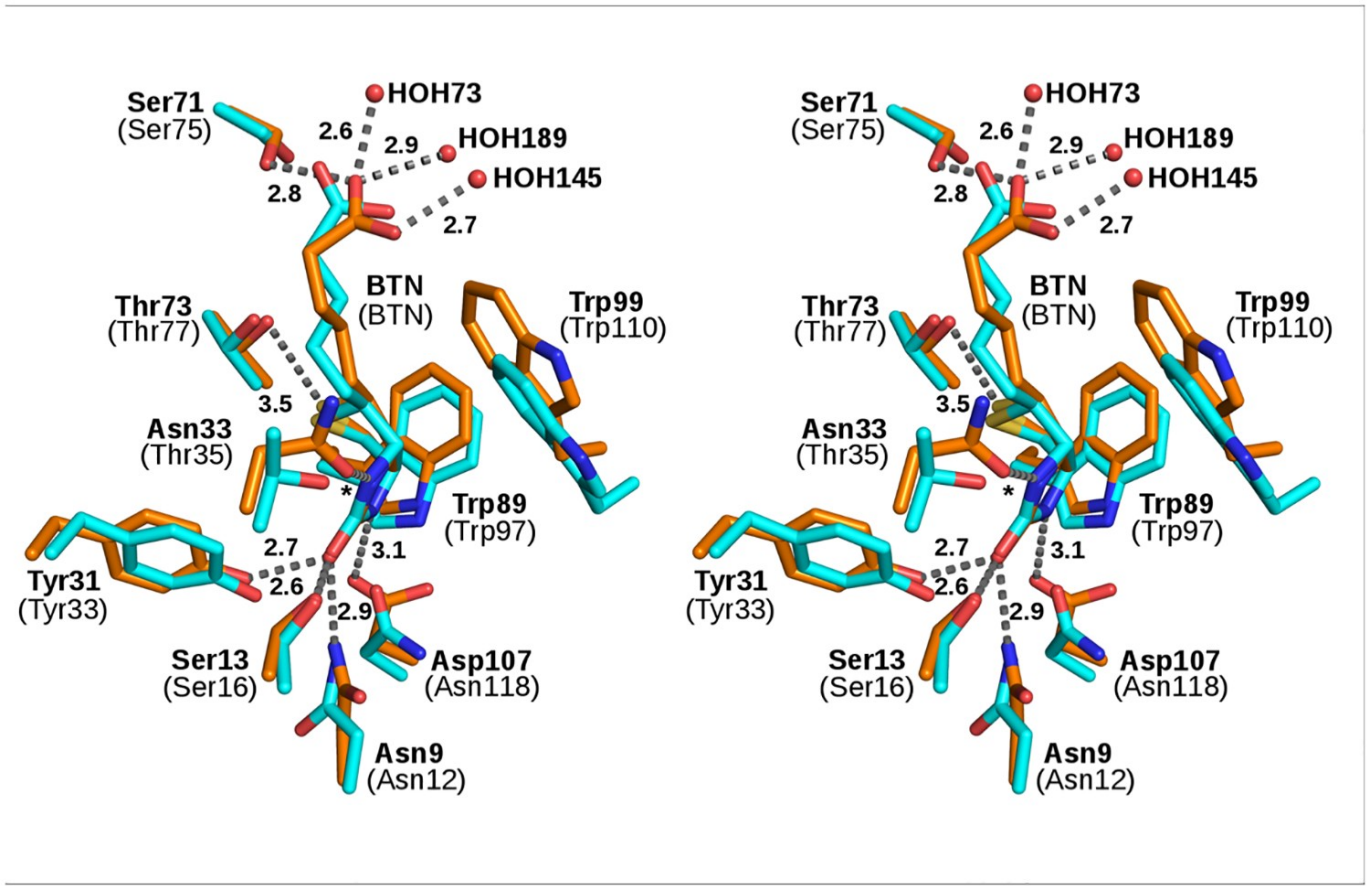
Comparison (stereo view) of the biotin-binding residues (sticks) of core-bradavidin (orange; bold labels) [PDB:4BBO] and chicken Avd (cyan; labels in brackets) [PDB:1AVD]. The Cα traces were superimposed. Trp99 (Trp110 in Avd) is shown from subunit III; other residues are from subunit I. The bound biotin ligands are drawn as thick sticks. Nitrogen atoms are shown in blue, oxygen atoms in red and sulfur atoms in yellow. Water molecules are drawn as red spheres and H-bonds for core-bradavidin as grey dashed lines (distances in Ångströms; * = 2.8 Å).

Despite the low sequence identity between core-bradavidin and chicken Avd, the deeply buried residues involved in biotin binding and the mode of binding are highly conserved (Fig 2). Like in chicken Avd [PDB:1AVD], the core-bradavidin–biotin interaction is stabilized by several hydrogen bonds (H-bonds) (Fig 2). In more detail, 1) Asn9 Nδ (Asn12 in Avd), Ser13 Oγ (Ser16) and Tyr31 Oη (Tyr33) all form H-bonds with the 2´ oxygen atom of the ureido ring of biotin; 2) Asp107 Oδ (Asn118) forms a H-bond interaction with the 1´ nitrogen atom of the ureido ring; 3) Asn33 Oδ (Thr35) forms a H-bond to the 3´ N atom of the ureido ring; 4) Thr77 Oγ (Thr77) has polar interactions with the sulfur atom of the tetrahydrothiophene ring; and 5) Ser75 Oγ (Ser75) forms a H-bond to one oxygen atom of the carboxylate group of the valeric acid moiety (bradavidin numbering according to [40]). Three structural water molecules (HOH73, HOH145 and HOH189) are also located close to the carboxylate end of biotin. Moreover, several conserved hydrophobic interactions typical for Avds are also seen in core-bradavidin and include the interaction of biotin with Trp89 (Trp97 in Avd) and Trp99 (Trp110; from another subunit); these residues are respectively 3.7 Å and 4.3 Å distant from biotin.

### Biotin-binding mode of core-bradavidin—Unique features

**Fig 3:**
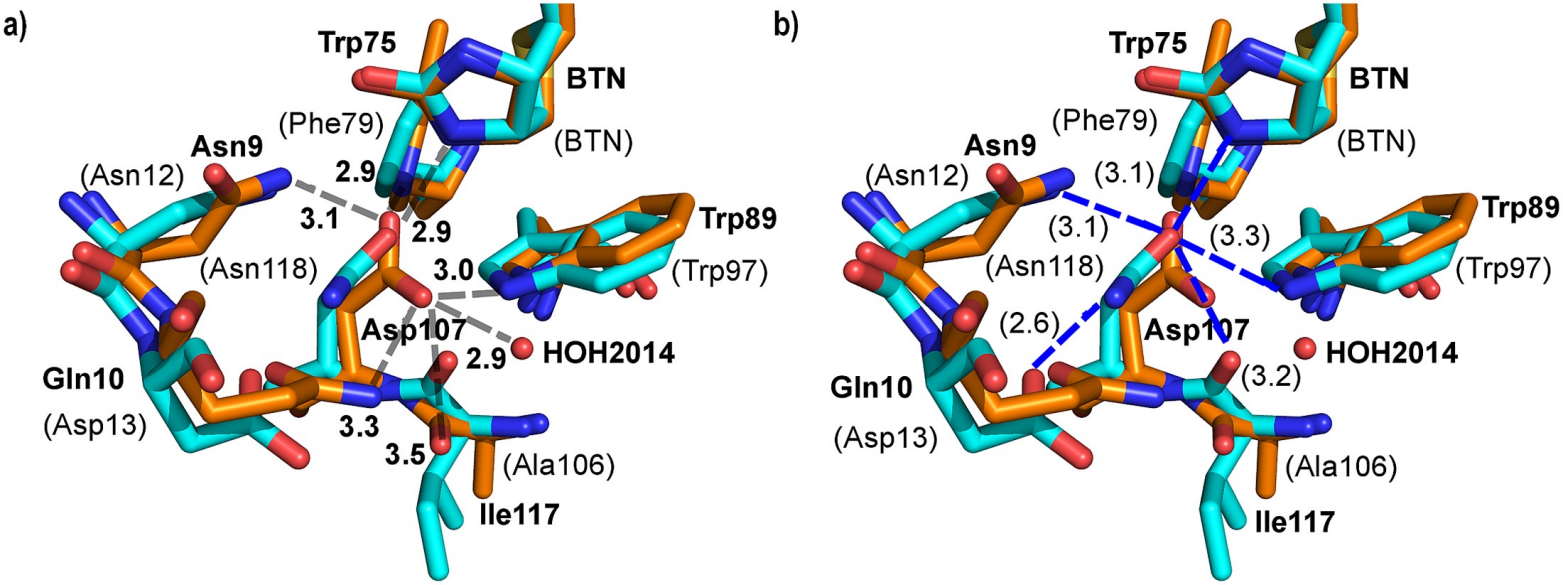
Interactions of Asp107 of core-bradavidin (orange; bold labels) and the equivalent Asn118 of chicken Avd (cyan; labels in brackets). Seven H-bonds stabilize the side chain of Asp107 in core-bradavidin (a), whereas only five H-bonds stabilize the equivalent Asn118 in chicken Avd (b). Non-carbon atom colouring as in Fig 2. H-bonds are drawn as grey dashed lines for core-bradavidin (a) and blue dashed lines for chicken Avd (b); distances in Ångströms.

Asp107 and Asn33 of core-bradavidin are equivalent to Asn118 and Thr35 in Avd [PDB: 1AVD]. The side-chain oxygen atom of each of these residues interacts with the 1´ and 2´ N atoms of biotin but the overall H-bonding network of the side chains of these residues with the surrounding residues varies between core-bradavidin and Avd (Fig 3). Asp107 in core-bradavidin is within H-bonding distance of a structural water molecule (HOH2014), Asn9, Gln10, Trp75 (equivalent to Phe79 in Avd), Trp89 and Ala106, whereas in Avd only Asn12, Asp13, Trp97 and Ile117 are sufficiently close to the corresponding residue Asn118. Both sets of residues make hydrophobic interactions with the ureido ring moiety of biotin in core-bradavidin and in Avd.

In bradavidin Tyr11 may be of special importance for ligand binding, since in the structure of the biotin complex of core-bradavidin it has moved significantly in comparison to the location in the wt bradavidin structure (see below). This residue is also poorly conserved and, to our knowledge, a tyrosine residue at the equivalent position is only found in a few bacterial Avds, which includes the only other reported Brad-tag containing Avd, rhodavidin from *Rhodopseudomonas palustris* [39], and the novel Avd-like sequences that we have identified in the genera *Bradyrhozibium*, *Mesorhizobium*, *Burkholderia (Pseudomonas)*, *Catenulispora and Actinocatenispora* (data not shown).

**Fig 4:**
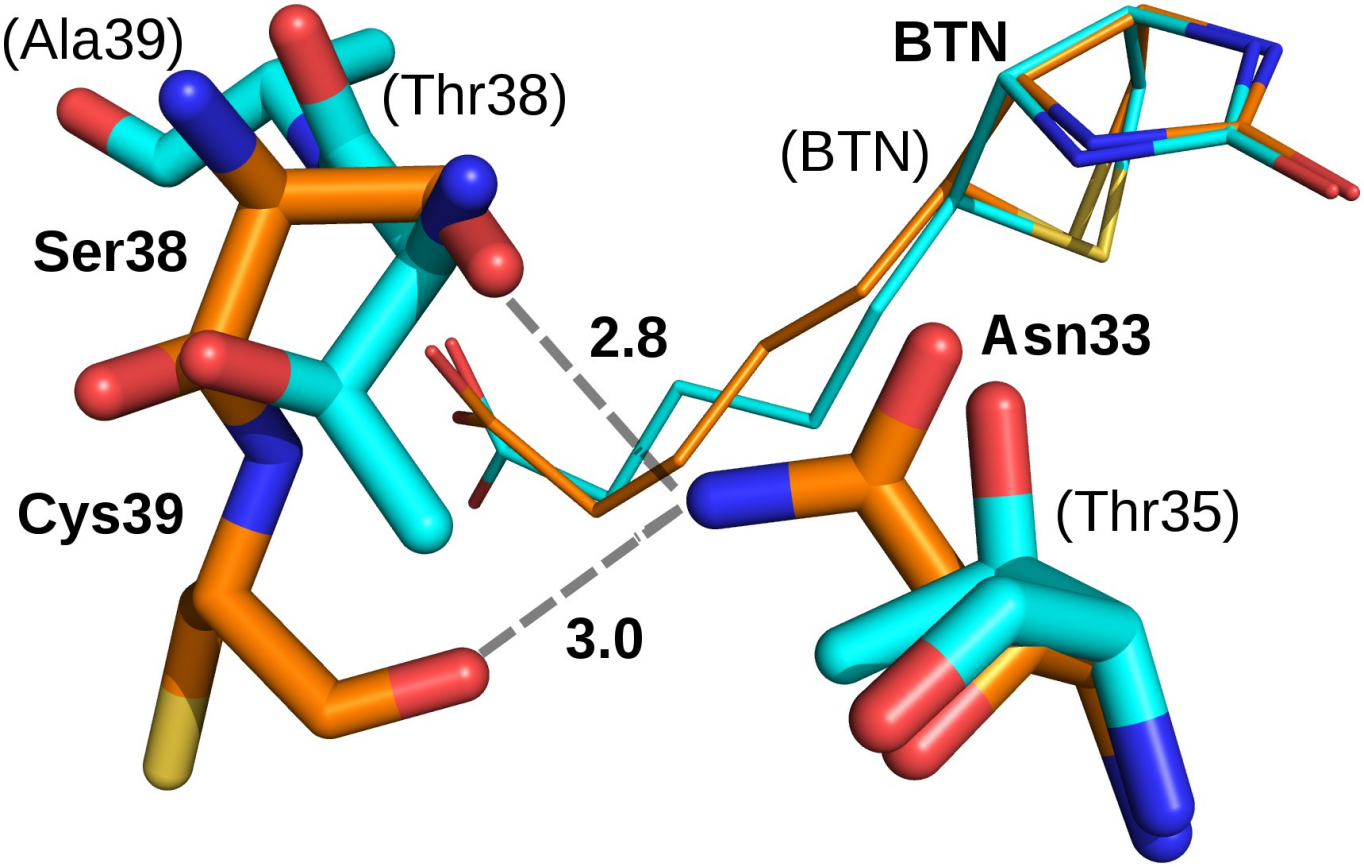
Interactions of Asn33 of core-bradavidin (orange; bold labels) and the equivalent Thr35 of chicken Avd (cyan; labels in brackets). Asn33 of core-bradavidin is H-bonded (grey dashed lines) to Ser38 and Cys39, whereas in chicken Avd the equivalent H-bonds cannot be formed. Biotin molecules for both proteins are shown as thin sticks. Non-carbon atoms are coloured as in Fig 2. Distances are shown in Ångströms.

**Fig 5:**
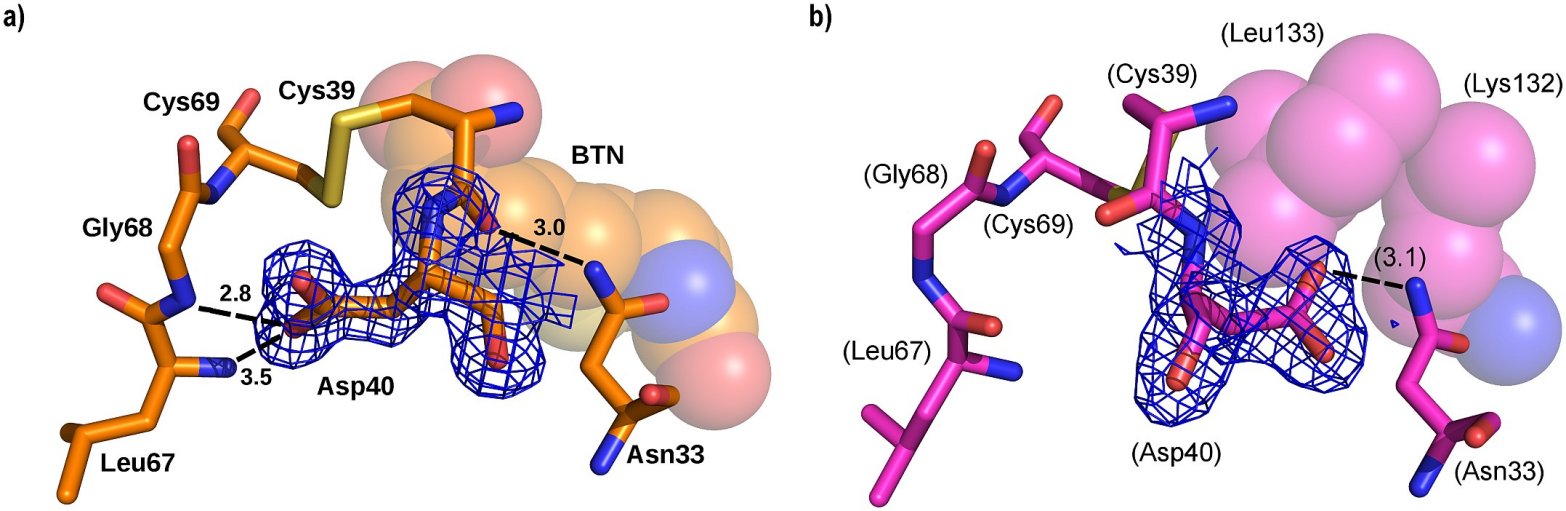
Comparison of Asp40 in core- (orange; bold labels) [PDB:4BBO] and wt bradavidin (magenta; labels in brackets) [PDB:2Y32]. In core-bradavidin (a), the side chain of Asp40 is flipped to an opposite direction as compared to wt bradavidin (b). Biotin (a) and residues K132 and L133 (b) occupying the same space as biotin in core-bradavidin (see a) are shown as spheres. Non-carbon atoms are coloured as in Fig 2. H-bonds are shown as dashed lines; distances in Ångströms. The weighted 2Fo-Fc electron density map around Asp40 (a, b) is shown as a blue mesh (contour level of 1.0 σ).

**Fig 6:**
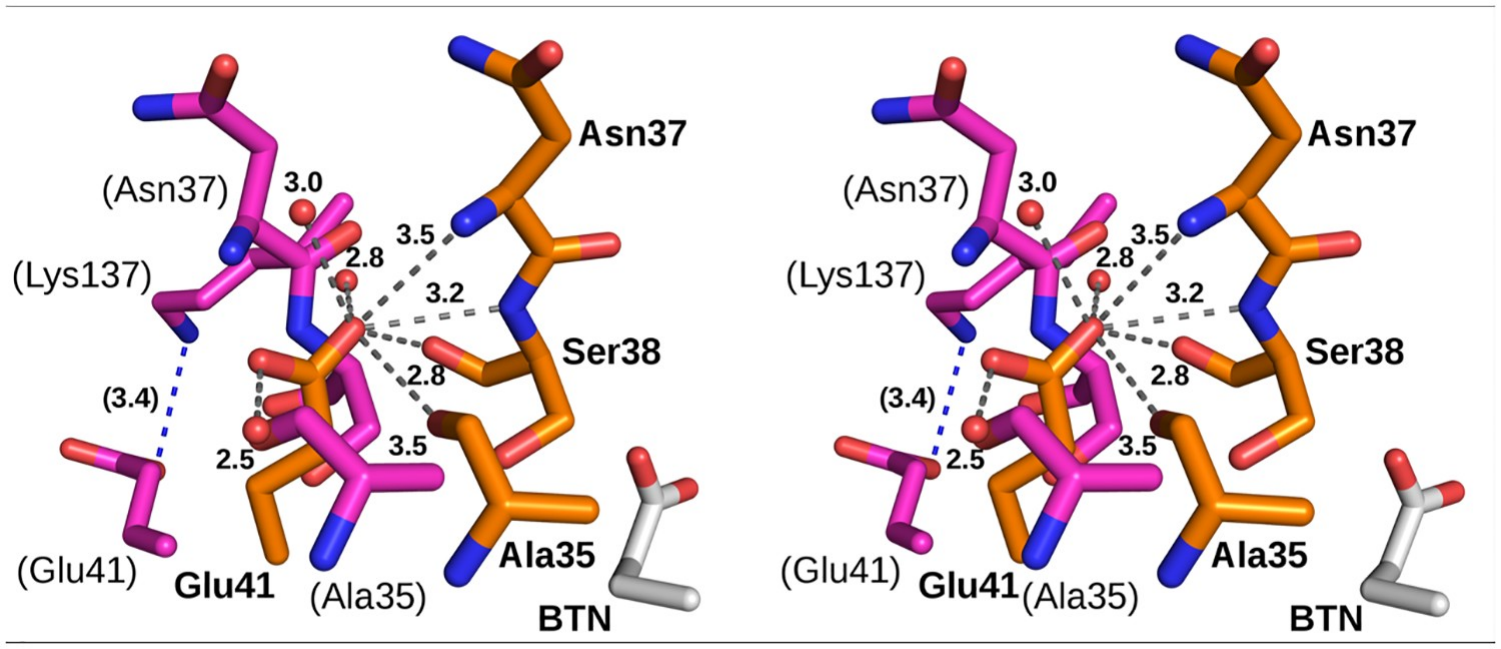
Comparison (stereo view) of Glu41 in core-bradavidin (orange; bold labels) [PDB:4BBO] and wt bradavidin (magenta; labels in brackets) [PDB:2Y32]. In core-bradavidin, the side chain of Glu41 is H-bonded (grey dashed lines) to several neighboring residues and is oriented in the opposite direction as compared to wt bradavidin, where the side chain of Glu41 is facing the solvent and H-bonded (blue dashed line) only to Lys137. A part of the acyl moiety of the bound biotin (BTN) of the core-bradavidin structure is shown as light grey sticks. Non-carbon atom colouring as in Fig 2. Water molecules are drawn as small red spheres. Distances are shown in Ångströms.

The Nδ atom of Asn33 in core-bradavidin is H-bonded to Ser38 Oγ and Cys39 O (Fig 4), whereas in chicken Avd the equivalent H-bonds are missing. Out of the known crystal structures of other Avds, similar H-bond interactions are only seen in rhizavidin [PDB:3EW2] and hoefavidin [PDB:4Z28]. In both structures, an asparagine residue equivalent to Asn33 of core-bradavidin is stabilized by H-bonds to the side-chain oxygen atom of a threonine residue (Ser38 Oγ in core-bradavidin) and to the main-chain oxygen atom of a glycine residue (Cys39 O in core-bradavidin). In wt bradavidin, Asn33 is not connected to these residues but instead H-bonds to Asn33 Nδ and Asp40 Oδ, the orientation of the Asp40 side chain being flipped in the opposite direction in comparison to core-bradavidin due to a rearrangement of the L3,4 loop. Moreover, in core-bradavidin, the side chain of Asp40 is H-bonded to Leu67 N and Gly68 N of the L5,6 loop, to Lys43 Nζ of the L3,4 loop, and to Cys69 S (L5,6 loop) that forms the disulfide bridge with Cys39 (Fig 5). The side-chain orientation of Glu41 is also flipped to the opposite direction in the core-bradavidin structure (closed L3,4 loop) *versus* the wt bradavidin structure (open L3,4 loop), and stabilized by different interactions (Fig 6). Hence, Asp40 and Glu41 may have a vital role for ligand binding and in stabilizing the unique L3,4 loop conformations of wt bradavidin (Brad-tag as ligand) and core-bradavidin (biotin as ligand), two variations of the same protein with quite different bound ligands.

In comparison with Trp99 in wt bradavidin, in core-bradavidin the tryptophan residue moves by 2 Å, enabling the residue to better seal the ligand-binding pocket containing biotin (see below). An Ångström-scale shift in atomic position is also seen for Ser38 of core-bradavidin [PDB:4BBO] in comparison to wt bradavidin [PDB:2Y32]. The movement of Ser38 mimics the “pinching effect” reported for the equivalent threonine residue in hoefavidin [21] [PDB:4Z6J, 4Z28] and rhizavidin [41] [PDB: 3EW1, 3EW2]; both Thr55 of hoefavidin and Thr48 of rhizavidin of the L3,4-loop respectively more closely approach Leu113 and Leu104 (located on the β7-strand; Leu91 in bradavidin) as a result of biotin binding (a similar “pinching effect” can also be seen in shwanavidin [20] [PDB:3SZH, 3SZJ] and bradavidin II [23] [PDB:4GGT, 4GGZ]). In each of these structures, the leucine side chains occupy the same relative locations, suggesting that the pinching effect might not be restricted only to dimeric Avds and, in general, might reflect an adaptation of the L3,4-loop for ligand binding.

The aromatic residues Trp70 and Phe79 of chicken Avd are respectively replaced by Phe66 and Trp75 in bradavidin. Even subtle differences such as these may have an effect on the flexibility of the L5,6 loop and the biotin-binding properties of core-bradavidin: in Avd, Trp70 Nη is H-bonded to Thr77 Oγ; whereas, in bradavidin, an equivalent H-bonding interaction is missing. To our knowledge, all of the bacterial Avds identified so far—including both dimeric and tetrameric proteins—have a tryptophan residue at the position equivalent to Trp75 of bradavidin, whereas all of the characterized eukaryotic Avds have a phenylalanine residue at this position.

### The disulfide bridge of bradavidin—Unusual configuration

**Fig 7:**
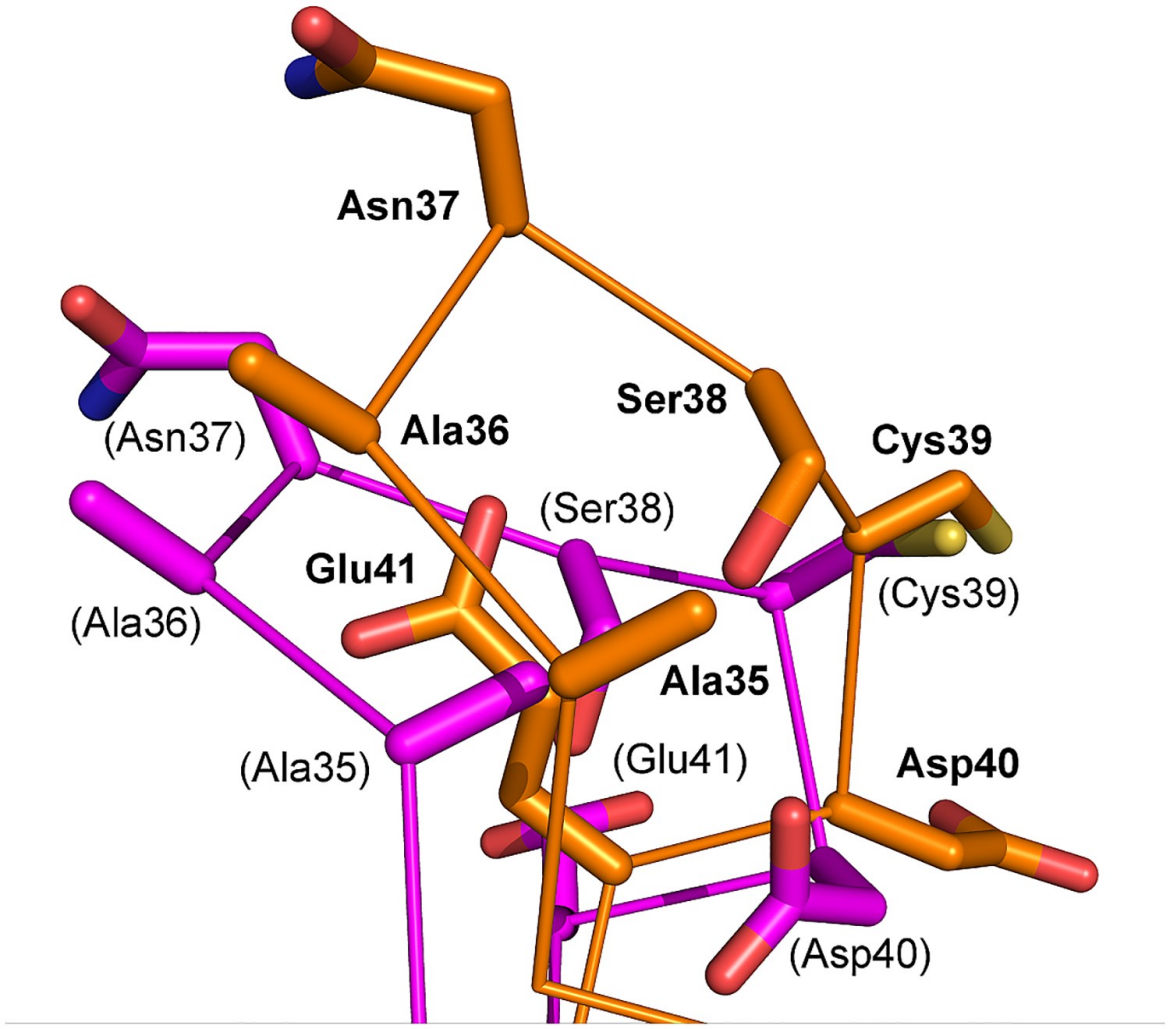
Superimposition of the residues 35–41 (L3,4 loop) of core-bradavidin (orange; bold labels) [PDB:4BBO] and wt bradavidin (magenta; labels in brackets) [PDB:2Y32].

The L3,4 loop of tetrameric core-bradavidin and of wt bradavidin is stabilized by a disulfide bridge between residues Cys39 (L3,4 loop) and Cys69 (L5,6 loop), similarly to dimeric rhizavidin [PDB:3EW2] [41], shwanavidin [PDB:3SZJ] [20] and hoefavidin [PDB:4Z28] [21], as well as bradavidin II [PDB:4GGZ] with its highly dynamic oligomeric structure [23]. In bradavidin, however, cysteine Cys39 of the L3,4 loop is located one residue earlier in the sequence, as reported recently by Avraham et al. (2015) for hoefavidin [21]. Thus, Cys39 is not exactly structurally equivalent to Cys50 in rhizavidin, Cys45 in shwanavidin, Cys57 in hoefavidin and Cyss44 in bradavidin II, whereas the other cysteine of the disulfide bond within the L5,6 loop, Cys69 in bradavidin, is conserved despite the fact that the conformation of the L5,6 loop varies within these proteins. Moreover, in the case of the biotin-complex structure of core-bradavidin, the earlier position of the cysteine residue does not seem to clearly affect the conformation of the L3,4 loop, which is in a similar conformation in all of the biotin complex structures listed above. In comparison to the wt bradavidin structure, which lacks biotin but has the Brad-tag sequence within the ligand-binding pocket, the configuration of the disulfide bridge of core-bradavidin is, however, altered and directly related to the position and conformation of Cys39 (Fig 7). As described above, the residue adjacent to Cys39 in bradavidin, Asp40, may have a special importance here, too, because the side chain is flipped in the opposite direction in core-bradavidin *versus* wt bradavidin. It is not yet known how the Brad-tag itself affects the conformation of the L3,4 loop when and if biotin were bound in the intact wt structure, since we have not been not able to crystallize wt bradavidin in complex with biotin. All in all, the configuration of the disulfide bond in both forms of bradavidin, core- with biotin and wt *sans* biotin, are unique, and likely do represent the unique structural features needed to enable the presence and recognition of two different ligands by the bradavidin structure.

### Subunit interfaces

Avds are stabile over a wide range of conditions, including temperature and pH. The core-bradavidin–biotin complex with T_m_ = 97.9±0.2°C is less stable than chicken Avd (T_m_ with bound biotin ≈ 118°C), streptavidin (T_m_ with bound biotin ≈ 112°C), [32] and even wt bradavidin (T_m_ without biotin = 96.2±0.1°C, and 101.7±0.1°C with biotin) [33]. The key interactions responsible for stability are found at the subunit interfaces, which can be divided into three major categories: the interface between subunits I and II (IF1,2), between subunits I and III (IF1,3) and between subunits I and IV (IF1,4 interface); in the dimeric Avds, only the IF1,4 interface is present.

All of the subunit-subunit interfaces of core-bradavidin and wt bradavidin are structurally highly similar. A key interfacial residue—Tyr90 from each of the four subunits—is located at the center of the tetramer in both forms of bradavidin, whereas in chicken Avd [PDB:1AVD] and streptavidin [PDB: 3YR2] the residues equivalent to Tyr90 are respectively Leu98 and Leu109. Since we have recently published the detailed analysis of the subunit interface of wt bradavidin [33], we will focus here only on regions unique to core-bradavidin as compared to wt bradavidin. At the IF1,2 interface of core-bradavidin, the position of Trp99 (subunit II; equivalent to Trp110 of chicken Avd) at the tip of the L7,8 loop and the spatial arrangement of Trp99 with respect to Tyr11 (subunit I) differ by over an Ångström in comparison to wt bradavidin (S1 Fig). These differences, together with the conformational adaptation of the L3,4 loop, are the major differences that help these bradavidin structures recognize two very different ligands, biotin and the Brad-tag. The C-terminal Brad-tag sequence enters the ligand-binding pocket of wt bradavidin between Tyr11 and Trp99, and Tyr11 Oη (subunit I) forms a H-bonding interaction with Ser130 N (3.4 Å; subunit III) of the Brad-tag sequence and with one structural water molecule (HOH2027). In core-bradavidin, Tyr11 Oη H-bonds to three water molecules (HOH2014, HOH2015 and HOH2017).

The core- of the IF1,3 interface in the core- and wt bradavidin structures is formed by residues Gln86, Leu88, Tyr90, Ala104 and Ala106, and is structurally highly similar. The IF1,4 interface is also very similar in both structures: it is clearly the largest interface in terms of contact area and the number of residues involved—47 in wt bradavidin [33]—and both the core- and wt bradavidin structures are stabilized by various non-covalent interactions as listed in [33]. The L7,8 loops, however, have different conformations since the residues Gly57-Tyr63 are in contact with and adapt to the binding of biotin to core-bradavidin and the Brad-tag to wt bradavidin.

### Effect of the C-terminal Brad-tag sequence for the fold of bradavidin

In wt bradavidin, the open conformation of the L3,4 loop accommodates the amino acids of the Brad-tag sequence [42], whereas in the biotin-complex structure of core-bradavidin the L3,4 loop adopts a closed conformation (Fig 7). The most dramatic differences are found in the coordinates of residues Ala35-Glu41 of the L3,4 loop and the conformation of the side chain of Tyr31 is also different in these structures. As mentioned above, the conformation of Cys39, and the position of its Cα atom, varies also between these two bradavidin structures even though Cys69, which pairs with Cys39, has the same conformation in both structures. The importance of the equivalent disulfide bridge for biotin binding to shwanavidin and rhizavidin has been recently demonstrated using mutagenesis analysis [20]. This disulfide bridge has also been suggested to be important for biotin binding in hoefavidin and, in general, for all dimeric Avds [21], where the disulfide bridge is considered to maintain the L3,4-loop in the closed conformation. Interestingly, several different crystal structures of hoefavidin were recently determined by Avraham et al. (2015), including intact hoefavidin with its C-terminal, intrinsic peptide at the ligand-binding site [PDB:4Z6J]; the short hoefavidin structures truncated at the C-terminus (missing 10 residues) with a bound peptide derived from the 12 C-terminal residues of the intact protein [PDB 4Z2P, 4Z2V, 4Z2O]; and the short hoefavidin structures with [PDB:4Z28] and without [PDB:4Z27] a bound biotin molecule. In each of these structures, the L3,4-loop is in the closed conformation and only subtle differences were seen in the conformation of the L3,4-loop among the hoefavidin structures [21]. In bradavidin, however, the closed conformation of the L3,4-loop is only seen in the biotin-complex structure with core-bradavidin; whereas, the L3,4-loop in the wt bradavidin structure is in open conformation, which is clearly different to what is seen in the hoefavidin structures, the biotin-complex structure of rhizavidin [PDB:3EW2] and the shwanavidin structure [PDB:3SZJ]. Thus, the disulfide bridge of bradavidin has unique features, not least because it is the only example stabilizing the L3,4-loop in a tetrameric Avd but also because of its apparent ability to enable the binding of ligands in both the closed and open conformation of the L3,4-loop, depending on ligand type.

The superimposition of the core- and wt bradavidin structures confirmed that the side chains of Lys132 and Leu133 in wt bradavidin occupy the space equivalent to that occupied by biotin in the core-bradavidin structure: Leu133 spatially matches the carboxylate end of the biotin, whereas Lys132 occupies the same coordinate space as the bicyclic ring moiety of biotin (Fig 5), well in line with what was predicted recently by [33]; the binding mode of Brad-tag is discussed in detail in [33].

### Core-bradavidin V1 and CC mutant

**Fig 8:**
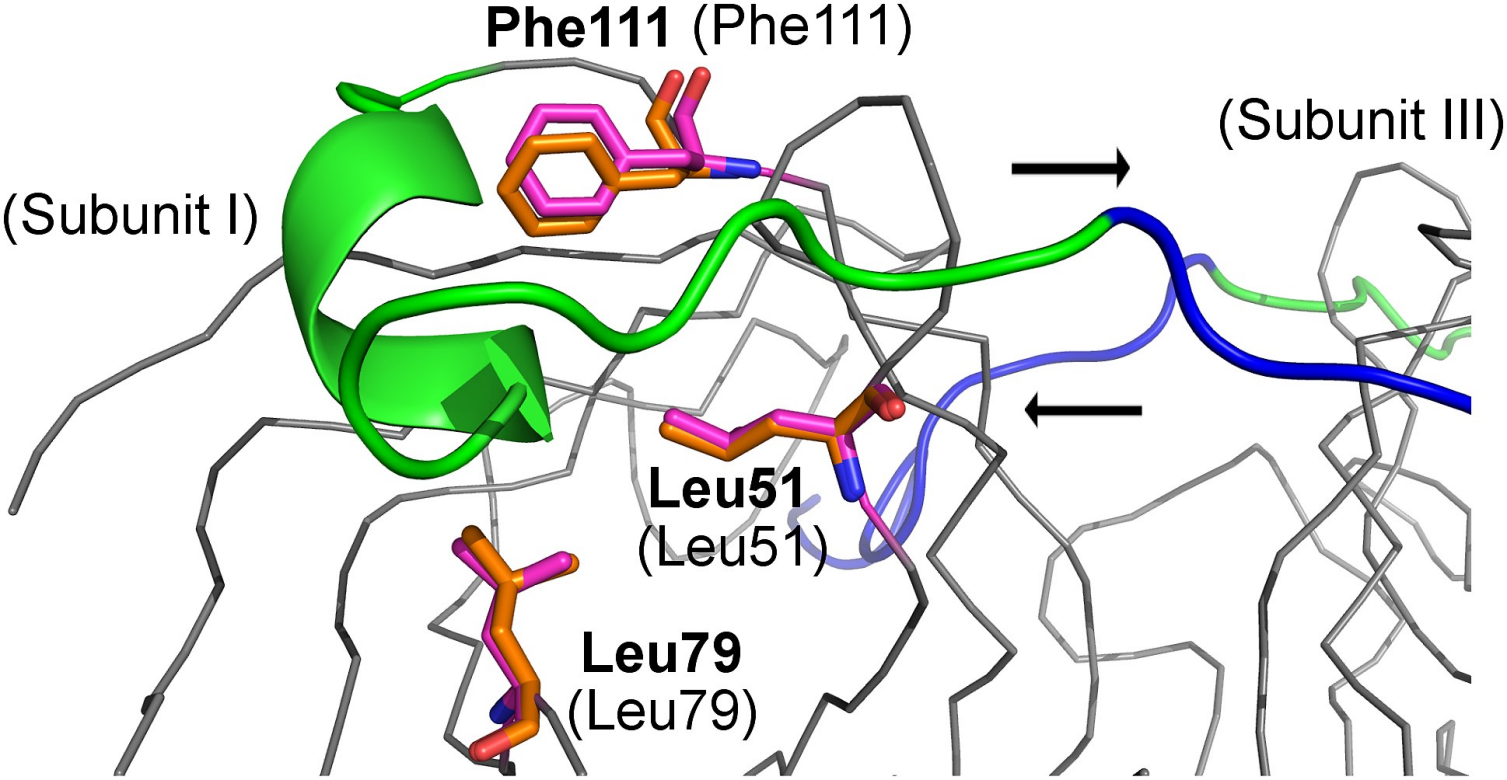
Illustration of the hydrophobic residues Leu51, Leu79 and Phe111 of wt bradavidin (magenta sticks, grey Cα trace; labels in brackets) [PDB:2Y32] that are exposed to solvent in the original core-bradavidin structure (orange sticks; bold labels) [PDB:4BBO] because of the lack of the C-terminal residues 114–127 (green/blue cartoon) of wt bradavidin. The core-bradavidin V1 construct extends to residues 115–127 (green) and lacks only residues 128–138 (blue), which enter into and occupy the ligand-binding pocket of a neighbouring subunit in the wt bradavidin structure. Arrows indicate the direction of the polypeptide chains: going to a neighbouring subunit (from subunit I to subunit III: →) and coming from a neighbouring subunit (from subunit III to subunit I: ←). Non-carbon atom coloring as in Fig 2.

Core-bradavidin is missing residues 114–138 of wt bradavidin. Of these residues, 114–127 fold onto the surface of the same subunit where they originated, residues 130–138 (Brad tag) interact with the ligand-binding site of another subunit (*e*.*g*., subunit I interacts with subunit III and *vice versa*) and Ala128-Gly129 form a linker that connects subunits I and III in wt bradavidin. In core-bradavidin, several hydrophobic residues, such as, Leu51, Leu79 and Phe111, become, at least partially, exposed to solvent as a result of the deletion of residues 114–138 of wt bradavidin (Fig 8). In wt bradavidin, the hydrophobic residues Leu51, Leu79 and Phe111 form a complementary fit with the side chain of Leu119. Whereas, in the core-bradavidin structure reported here, a glycerol molecule (subunit I), an acetate ion (subunits II, IV) and two water molecules (HOH2014, 2056) that are unusually close to each other (2.1 Å), are built into the position equivalent to Leu119 of wt bradavidin. These molecules/ions probably mask the hydrophobic surface that is exposed because of the lack of the C-terminal end of wt bradavidin in core-bradavidin. Therefore, a modified core-bradavidin form (core-bradavidin V1), having residues 1–127 but missing only residues 128–138 of wt bradavidin, was prepared here and expressed in *E*. *coli* with good yield (>10 mg/L). We were also interested to examine the role of the disulphide bridge in the vicinity of the ligand-binding site for the function of the protein. Therefore, a core-bradavidin mutant lacking the disulphide bridge (CC mutant) was produced by mutating Cys39 and Cys69 to alanine residues; unfortunately, the CC mutant was expressed with poor yield (~ 1 mg/L). The ligand-binding characteristics of core-bradavidin V1 and the CC mutant were then examined using fluorescently labelled biotin: the dissociation rate of the fluorescently labelled biotin was highly similar between core-bradavidin (k_diss_ = 1.0 ± 0.1 x 10^−4^ s^-1^ at 50°C), core-bradavidin V1 (k_diss_ = 1.2 ± 0.2 x 10^−4^ s^-1^ at 50°C) and the CC mutant (k_diss_ = 1.5 ± 0.4 x 10^−4^ s^-1^ at 50°C). This indicates that the disulphide bridge is not important for the binding of fluorescently conjugated biotin. Moreover, a similar thermal stability was observed: the T_m_-values with and without biotin being 73.2±0.3°C and 97.9±0.2°C, respectively, for core-bradavidin, and 73.5±0.5°C and 96.3±0.1°C for core-bradavidin V1. Core-bradavidin V1 (K_d_ = 2.2 ± 0.2 x 10^−5^ M) shows similar exothermic binding to brad-tag as core-bradavidin (K_d_ = 2.6 ± 0.3 x 10^−5^ M) [33] at 40°C, measured using isothermal titration calorimetry (ITC) as described in [33].

### Homology models of rhodavidin and an avidin from Bradyrhizobium sp. Ai1a-2

Rhodavidin is an avidin-like protein from *Rhodopseudomonas palustris*, a photosynthetic bacterium found mostly in swine waste lagoons [39]. Based on sequence analysis, rhodavidin (Uniprot: Q218I6; GenBank: WP_011472104.1) and an avidin-like protein from Bradyrhizobium sp. Ai1a-2 (bradavidin A2; Genbank: WP_051334960.1 has replaced GenBank record WP_027584113.1 that was removed from the GenBank database during the course of this study) also have Brad-tag sequences at their C-termini. In order to better understand whether the Brad-tag-binding mode is conserved in bradavidin, rhodavidin and bradavidin A2, and since no X-ray structures of rhodavidin and bradavidin A2 were available, we used homology modeling to construct the 3D structures of rhodavidin and bradavidin A2 (S2 Fig).

As expected based on the high similarity between the amino acid sequences of wt bradavidin and rhodavidin (75% identity), and between wt bradavidin and bradavidin A2 (74% identity), the homology models of rhodavidin and bradavidin A2 are very similar to the X-ray structure of wt bradavidin. The major differences are found at the residues just before the Brad-tag sequence. In the model of rhodavidin these residues—Lys128-Thr132 –extend the linker region located before the Brad-tag sequence and are not likely to directly affect the binding of the Brad-tag. However, the side chain of a tyrosine residue from the neighboring subunit (equivalent to Tyr11 of subunit I in wt bradavidin) moves slightly to accommodate Thr132 and Gly133 (the first residue of Brad-tag in wt bradavidin, subunit III). Similarly to the rhodavidin model structure, Ala127-Gly130 also extend the linker located just before the Brad-tag matching residues in bradavidin A2. However, in bradavidin A2 the first two residues, Ala131 (Gly129 in wt bradavidin) and Gly132 (Ser130), of the Brad-tag differ and may have an influence on the ligand binding affinity. Moreover, even though the subunit interfaces of both rhodavidin and bradavidin A2 seem to have a similar architecture to wt bradavidin, both rhodavidin and bradavidin A2 have a leucine instead of a tyrosine residue (Tyr90 in wt bradavidin) at the interface of the four subunits. Since the extremely tight biotin binders and high stability Avds, chicken Avd and streptavidin, also have a leucine residue at the subunit interface, rhodavidin and bradavidin A2 may exhibit higher stability than wt bradavidin. For reference, bradavidin II, showing dynamic oligomeric state, bears threonine in this position [43].

## Discussion

Chicken Avd [1] and streptavidin [5] have been extensively studied and used for various applications, including affinity purification and molecular labeling, because of their tight binding to biotin or its analogs [1]. However, certain Avds, such as bradavidin [32] and streptavidin [16], and the recently discovered hoefavidin [21], are known to bind ligands other than biotin, *i*.*e*. intrinsic peptide ligands. Our interest is to study and explore the possibility of utilizing core-bradavidin–Brad-tag pair in biotechnology.

Here, we reported the X-ray structure of core-bradavidin—biotin complex at 1.60 Å resolution and compared it to the known structures of Avds, including, *e*.*g*., the tetrameric chicken Avd, as well as the dimeric rhizavidin and shwanavidin. In particular, we were interested in analyzing the differences between the structures of wt bradavidin with its intrinsic Brad-tag ligand and core-bradavidin with the bound biotin ligand. Since the mechanism through which biotin replaces the Brad-tag in wt bradavidin and the subsequent conformation of the replaced Brad-tag–tail in solution is still not known, we also tried to crystallize wt bradavidin in complex with biotin, but did not obtain any crystals. Recently, Avraham et al. (2015) [21] were also unable to co-crystallize biotin and intact hoefavidin, another recently reported, intrinsic peptide-ligand containing bacterial Avd.

Interestingly, core-bradavidin and wt bradavidin have a disulphide bridge between Cys39 of the L3,4 loop and Cys69 of the L5,6 loop, a bridge that typically stabilizes the “open-to-solvent” ligand-binding site of dimeric Avds. However, in tetrameric Avds, a tryptophan residue (Trp110 in chicken Avd) of the L7,8 loop from the adjacent subunit “seals” the ligand-binding pocket during biotin binding, whereas in dimeric Avds no such residue exists and, hence, the ligand-binding pocket is much more open to solvent in dimeric Avds in comparison to tetrameric Avds. Moreover, the geometry of the ligand-associated disulphide bridge is identical in all dimeric Avds, whereas a unique conformation is observed in bradavidin, the only tetrameric Avd with such feature; the conformation of Cys39 varies even between the core-bradavidin and wt bradavidin structures. Therefore, it is tempting to suggest that the disulphide bridge between Cys39 and Cys69 has a special purpose for wt bradavidin, perhaps to enable or facilitate the adaptation of the L3,4 loop for binding of two very different ligands, Brad-tag and biotin. However, binding analyses of the CC mutant with fluorescently labeled biotin revealed a similar dissociation rate to that observed for core-bradavidin. It is also possible that the disulphide bridge is important for protein folding, which could also explain the poor protein yield in the case of the CC mutant.

Apart from the disulphide bridge, its neighboring residues—Asp40 and Glu41—are also involved in the stabilization of the L3,4 loop region in core-bradavidin; in core-bradavidin the side chains of Asp40 and Glu41 flip in the opposite direction from that seen in wt bradavidin and form unique interactions, indicating that significant changes in the conformations of these residues have to take place in order to accommodate biotin *versus* a peptide ligand. Changes in the conformation and the spatial location of Trp99, Tyr11 and Leu91 (core- *versus* wt bradavidin) also indicate the protein’s adjustments required to adapt to two ligands with immensely different sizes and shapes. What conformations these residues would have in a wt bradavidin-biotin complex structure, is still an open question.

In a competitive binding assay the affinity of the Brad-tag for core-bradavidin is much less in comparison to the affinity of biotin [33]. In the current study, we tried to increase the binding affinity of Brad-tag to core-bradavidin through rational mutagenesis of the original Brad-tag peptide, but none of the mutant peptides led to increased affinity for core-bradavidin based on the DSC (differential scanning calorimetry) measurements (data not shown). To increase the affinity of the original Brad-tag, or variants of it, mutagenesis of core-bradavidin may be needed. Moreover, the homology models of rhodavidin and bradavidin A2 suggest that the very first residues of the Brad-tag sequence may affect Brad-tag binding; mutagenesis analysis of these residues may give ideas on how to further increase the affinity of Brad-tag to core-bradavidin.

From an evolutionary point-of-view, it would be interesting to know what is the exact function of the C-terminal Brad-tag sequence for bacterial Avds and what is the added value for the host organisms to have Brad-tag–containing Avds. Biotin is a cofactor needed by several carboxylases in enzymatic reactions important for the synthesis of fatty acids and the amino acids valine and isoleucine; biotin is needed also for gluconeogenesis [44]. It could be that, for example in *B*. *diazoefficiens*, bradavidin controls the availability of biotin through a regulating mechanism of its C-terminal Brad-tag extension. Interestingly, the soybean-*Bradyrhizobium* symbiosis is utilized for growing soybean in tropics because the symbiosis can be highly efficient in fixing nitrogen [45]. Could bradavidin have a role here? Further experiments are needed to address these questions.

## Materials and methods

### Crystallization and data collection

Core-bradavidin (approximately 1 mg/ml; 50 mM sodium acetate, 100 mM sodium chloride, pH 4) was crystallized using the vapour diffusion method. Sitting drops (1 μl of protein-biotin solution + 1 μl of well solution) were manually prepared. The protein-biotin solution was mixed using a ratio of 25 μL protein solution and 1 μL of biotin solution (1 mg/ml; 5 mM Tris pH 8.8 and 8 mM CHES pH 9.5), and incubated at +37°C for 3.5 hours before crystallization. The well solution used was derived from the commercial crystallization screen (Crystal Screen I; Hampton Research) and had 0.1 M HEPES pH 7.4, 0.8 M K/Na tartrate. For data collection, 0.7 μL of cryoprotectant (100% glycerol) was added to the crystallization drop just prior to freezing in liquid nitrogen. Data were collected at MAX-LAB beam line I711, Lund (Table 1). Data were processed using XDS [46].

**Table 1:** Structure determination statistics for core-bradavidin [PDB:4BBO].

DATA PROCESSING^a^	
Space group	*P*2_1_2_1_2_1_
Unit cell:	
a, b, c, (Å)	49.9, 78.6, 100.1
α, β, γ (°)	90, 90, 90
Wavelength (Å)	1.063
Beamline	MAX-LAB 1711, LUND
Resolution (Å)^b^	24.23–1.60 (1.70–1.60)
Observed reflections^b^	507744 (82930)
Unique reflections^b^	52798 (8651)
I/sigma^b^	17.8 (4.4)
*R*_factor_ (%)^b^	9.2 (55.3)
Completeness^b^	99.9 (100)
REFINEMENT	
*R*_work_ (%)^c^	14.4%
*R*_free_ (%)^c^	17.8%
Monomers per asymmetric unit	4
*R*.*m*.*s*.*d*:	
Bond lengths (Å)	0.015
Bond angles (°)	1.58

^a^The numbers in parenthesis refer to the highest resolution bin;

^b^Data from XDS;

^c^Data from Refmac 5.

### X-ray structure determination

Initial phase estimates for the structure factors were obtained using the molecular replacement program Phaser [47] within the CCP4i GUI [48,49]. For molecular replacement, a tetramer of wt bradavidin [PDB:2Y32] (residues 2–119 of each chain A-D) was used as the search model. The space group of the structure was confirmed to be *P*2_1_2_1_2_1_ after the replacement. The initial X-ray structure of the core-bradavidin was refined with Refmac5 [50] yielding an R_factor_ of 0.305, R_free_ of 0.332 and FOM (Figure of merit) = 0.755 and then manually edited/rebuilt using Coot [51], including the addition of water and biotin molecules. Solvent atoms and other heteroatoms were also added using ARP/wARP [52–55]. A few cycles of refinement in the middle of the structure building process were done with the software suite Phenix [56–58].

The final structure was validated by using the inbuilt tools of Coot [51] and MolProbity [59] of the Phenix suite [56–58]. PyMOL [60,61] and Bodil [62] were used to check the final structure, too. The final structure coordinates and structure factors were deposited into Protein Data Bank [63,64] with the PDB code 4BBO. The data collection and structure determination statistics are provided in Table 1.

### Search for sequences homologous to wt bradavidin

The NCBI sequence database (http://www.ncbi.nlm.nih.gov/) and the UniProt database (http://www.uniprot.org/) [65] were searched for Brad-tag–containing novel Avds using the program BLAST [66] and the sequence of wt bradavidin [PDB:2Y32] [33]. The sequence of rhodavidin from *Rhodopseudomonass palustris* (strain BisB18) was obtained from the Uniprot database (UNIPROT ID: Q218I6) [65] and was already described earlier in [33]. In addition to rhodavidin, a number of bradavidin-like sequences containing the Brad-tag sequence at their C-terminus and belonging to the *Bradyrhizobium* genus were found from the NCBI database. These included *Bradyrhizobium sp*. WSM1253 (Genbank: WP_007602433.1), *Bradyrhizobium sp*. Cp5.3 (Genbank: WP_051311274.1), *Bradyrhizobium sp*. th.b2 (Genbank: WP_035978291.1), *Bradyrhizobium sp*. Ai1a-2 (Genbank: WP_051334960.1), *Bradyrhizobium sp*. WSM2254 (Genbank: WP_049823387.1), *Bradyrhizobium sp*. WSM3983 (Genbank: WP_027532948.1) and *Bradyrhizobium sp*. Tv2a-2 (Genbank: WP_024521026.1).

### Homology modeling

The tetrameric 3D homology models were calculated using the crystal structure of wt bradavidin [PDB: 2Y32] as the template structure and using a structure-based sequence alignment (S3 Fig), which was done using the program Malign [67] within Bodil [62]. The alignment was used as an input for model building, which was done using Modeller 9.14 [68] with default parameters.

A total of 10 homology models were created and arranged based on their Modeller objective function (molpdf) score in ascending order. All the models were next validated for stereochemical and geometrical parameters using MolProbity [59]. The rhodavidin model selected for further analysis had 97.7% of the residues (547/560) in the most favoured regions and an additional 1.8% of residues (10/560) in allowed regions in Ramachandran plot; 0.5% (Ala127, chain A; Ser130, chain B; and Ile129, chain C) of the residues were outliers. The Cα atoms of the rhodavidin model superimposed to the wt bradavidin structure (chain A) with a root mean square deviation (RMSD) value of 2.2 Å.

Similarly, 10 homology models were created for bradavidin A2, and were tested and validated using MolProbity [59]. The bradavidin A2 model selected for further analysis had 97.7% of the residues (547/560) in the most favoured regions and an additional 2.3% (13/560) in additionally allowed regions in Ramachandran plot, with no outliers. After superimposing the wt bradavidin and bradavidin A2 structures, the RMSD value for the Cα atoms was 3.4 Å.

### Production of core-bradavidin V1 and CC mutant

The DNA sequences coding for core-bradavidin V1 and the CC mutant with N-terminal 6xHis (HHHHHH) and 3xFLAG tag (DYKDHDGDYKDHDIDYKDDDDK) inserted between the N-terminal signal peptide (MRHFNGMLLAMIASTSLIGPLPAYA; predicted to fully be cleaved off) and the mature proteins (core-bradavidin V1; QSV…DLK, CC mutant; QSV…C39A,C69A…KAL) were ordered as synthetic genes (Thermo Fisher Scientific, GeneArt) and subcloned into pET101/D-TOPO^®^ vector (Thermo Fisher Scientific) according to the manufacturer’s instructions. Sequences were confirmed (ABI PRISM 3100 Genetic Analyzer, Applied Biosystems), and clones were transformed with heat shock to *E*. *coli* BL21-AI cells (Thermo Fisher Scientific), which are suitable for production of toxic proteins due to the tight regulation of protein expression. Core-bradavidin V1 and CC variants were produced within the periplasmic space of *E*. *coli* BL21-AI cells in an active form, as previously described in detail [69]. Individual colonies were first cultured overnight in 5 ml volume of Lysogeny broth (LB) medium supplemented with 100 μg/ml ampicillin, 5 μg/ml tetracyclin and 0.1% (w/v) glucose, before the cells were diluted to 500 ml of LB with the same supplements. The growth of bacteria was followed by measuring the optical density (OD, absorbance at 600 nm wavelength) and, when the value of OD reached 0.4, induction was performed by adding 1 mM IPTG and 0.2% (w/v) L-arabinose. After overnight incubation at 28°C and horizontal shaking (200 rpm), the bacterial cells were harvested by centrifugation (10 minutes, 5000 g).

Proteins were purified in a single step using 2-iminobiotin affinity chromatography as described earlier in [33] for core-bradavidin. The isolated *E*. *coli* cells were suspended in binding buffer (50 mM Na-carbonate, 1 M NaCl, pH 11) after which the cell suspension was homogenized twice using an EmulsiFlex C3 homogenizator (Avestin Inc., Ottawa, Canada) and clarified by centrifugation (15 000 g, 30 min, 4°C). The pH of the crude protein mixture was adjusted to 10.5 using 10 M NaOH before it was applied to 2-iminobiotin agarose equilibrated with the binding buffer. The crude protein–agarose mixture was incubated for one hour on a rolling shaker at 4°C and the agarose was collected using centrifugation (3000 g, 10 min, RT). The agarose was then washed twice with the binding buffer and transferred to a column, where the protein was eluted in 1 ml fractions with 0.5 M acetic acid (pH 3). The purity of the proteins were analyzed using SDS-PAGE (15%) in reducing conditions. In addition, the protein concentration was determined with a UV/Vis spectrophotometer (NanoDrop 1000 Spectrophotometer, Thermo Scientific, Wilmington, DE, USA) by measuring the absorbance at 280 nm and using an extinction coefficient of 43555 M^–1^cm^–1^ and 43430 for core-bradavidin V1 and CC mutant, accordingly.

### Biophysical analysis of core-bradavidin V1 & CC mutant

The unfolding temperature of core-bradavidin V1 was analyzed using the VP-Capillary DSC instrument (GE Healthcare, MicroCal, Northampton, MA, USA) in 50 mM sodium phosphate buffer (150 mM NaCl, pH 7.2) with protein concentration of 0.2 mg/ml. Solutions were degassed prior to measurements. Samples were heated from 20°C to 130°C at a scanning rate of 2°C/min. Feedback mode was set to ‘low’ and the filter period was 5 s. The temperature transition midpoint (T_m_) was obtained from the midpoint of the curve that was fitted to the data after first subtracting the baseline from the measurement data and then using the Levenberg-Marquardt non-linear least-squares method to fit the curve using the MicroCal Origin 7.0 software (MicroCal, Malvern Instrument Ltd). Similar analysis was not possible with the CC mutant due to lack of protein for proper analysis.

The dissociation rate constant (k_diss_) of fluorescently labelled biotin was determined by fluorescence spectrometry using the biotin-labelled fluorescent probe ArcDia^™^ BF560 as described in [69]. In practice, 50 nM dye in a buffer containing 50 mM sodium phosphate, 650 mM NaCl and 0.1 mg/ml BSA (pH 7) was mixed with 100 nM core-bradavidin V1 (or CC mutant) and the change in fluorescence intensity was measured over time. A 100-fold molar excess of free biotin (D-biotin, Sigma-Aldrich Co. LLC., St. Louis, MO, USA) was used to monitor the dissociation of this complex. The assay was performed at 50°C using a QuantaMaster^™^ Spectrofluorometer (Photon Technology International, Inc., Lawrenceville, NJ, USA). Biotinylated BF560 was excited at 560 nm, and emission was measured at 578 nm.

The affinity of core-bradavidin V1 towards Brad-tag (peptide SEKLSNTK; GenScript, Piscataway, NJ, USA) was measured by ITC. The purified core-bradavidin V1 was dialyzed against 50 mM sodium phosphate (pH 7.0) buffer containing 100 mM NaCl, Brad-tag was dissolved in the same buffer and the samples were degassed using MicroCal^™^ ThermoVac. The analysis was performed at 40°C using an isothermal titration calorimetry VP-ITC MicroCalorimeter (GE Healthcare, MicroCal, Northampton, MA, USA) with 10 μl titration aliquots of Brad-tag in 30 repeated additions at intervals of 200 s using constant stirring speed of 440 rpm. The data were analyzed with Microcal Origin 7.0 (MicroCal LLC, Northampton, MA, USA) software. The observed reaction heats were corrected by subtracting the heat of dilution caused by the titration of the ligand alone into buffer. K_a_, ΔH and n (stoichiometry per subunit) were obtained through non-linear least-squares fit of the corrected reaction heats for each titration step.

### Miscellaneous methods

PyMOL [60,61] and Bodil [62] were used for analyzing structures, visualization and for creating figures. The structure-based sequence alignment was done by Malign [67] of the Bodil software package for biomolecular visualization and modeling [62]. A cut-off distance of ≤ 3.5 Å between non-hydrogen atoms was used for hydrogen bonds. Subunit one was used to create all figures unless not otherwise specified in the figure legends.

## Supporting information

S1 Fig Comparison of the subunit IF1,2 interface residues Trp99, Leu91 and Tyr11 of core-bradavidin (orange; bold labels) [PDB:4BBO] and wt bradavidin (magenta; labels in brackets) [PDB:2Y32] based on superimposition of the Cα traces of the proteins. The biotin molecule (BTN) of the core-bradavidin structure and the side chains of Trp99, Leu91 and Tyr11 are shown as sticks. Nitrogen atoms are coloured blue, oxygen atoms red and sulphur atoms yellow.
(TIF)

S2 Fig Comparison of the tertiary structure of wt bradavidin [PDB:2Y32] to the homology models of rhodavidin (Uniprot: Q218I6; Genbank: WP_011472104.1) and bradavidin A2 (Genbank: WP_051334960.1). Superimposition of the Cα traces of subunit I and subunit III of wt bradavidin (magenta), rhodavidin (blue) and bradavidin A2 (grey) are shown. The loop regions L1,2 to L7,8, and the N and C termini, are labelled.
(TIF)

S3 Fig Structure-based sequence alignment of core-bradavidin, chicken Avd, rhizavidin, shwanavidin, bradavidin II, hoefavidin, wt bradavidin, rhodavidin and bradavidin A2. The biotin-binding residues (top six structures) are marked with black squared boxes; the blue squared boxes indicate cysteine residues forming disulphide bridges in non-tetrameric Avds; the green ‘1’ indicates the cysteine residues forming disulphide bridges in bradavidins and rhodavidin; the black triangle indicates the tryptophan residue in equivalent position to Trp99 of the core-bradavidin structure that is present only in tetrameric Avds; and the ‘Brad-tag’ residues are highlighted with yellow background. The beta-strands 1–8 of core-bradavidin are labeled and indicated by arrows. The conserved residues are coloured by the default scheme of the ESPript 3 program (http://espript.ibcp.fr/ESPript/ESPript/).
(TIF)

S1 Table Yields of core-bradavidin, core-bradavidin V1 and CC mutant produced in *E*. *coli* BL21-AI. (DOCX)
