# Supplementary figures and images for: Structural characterization of core-bradavidin in complex with biotin
Source: PLoS One. 2017 Apr 20;12(4):e0176086. doi: 10.1371/journal.pone.0176086 (PMC5398887; doi:10.1371/journal.pone.0176086)

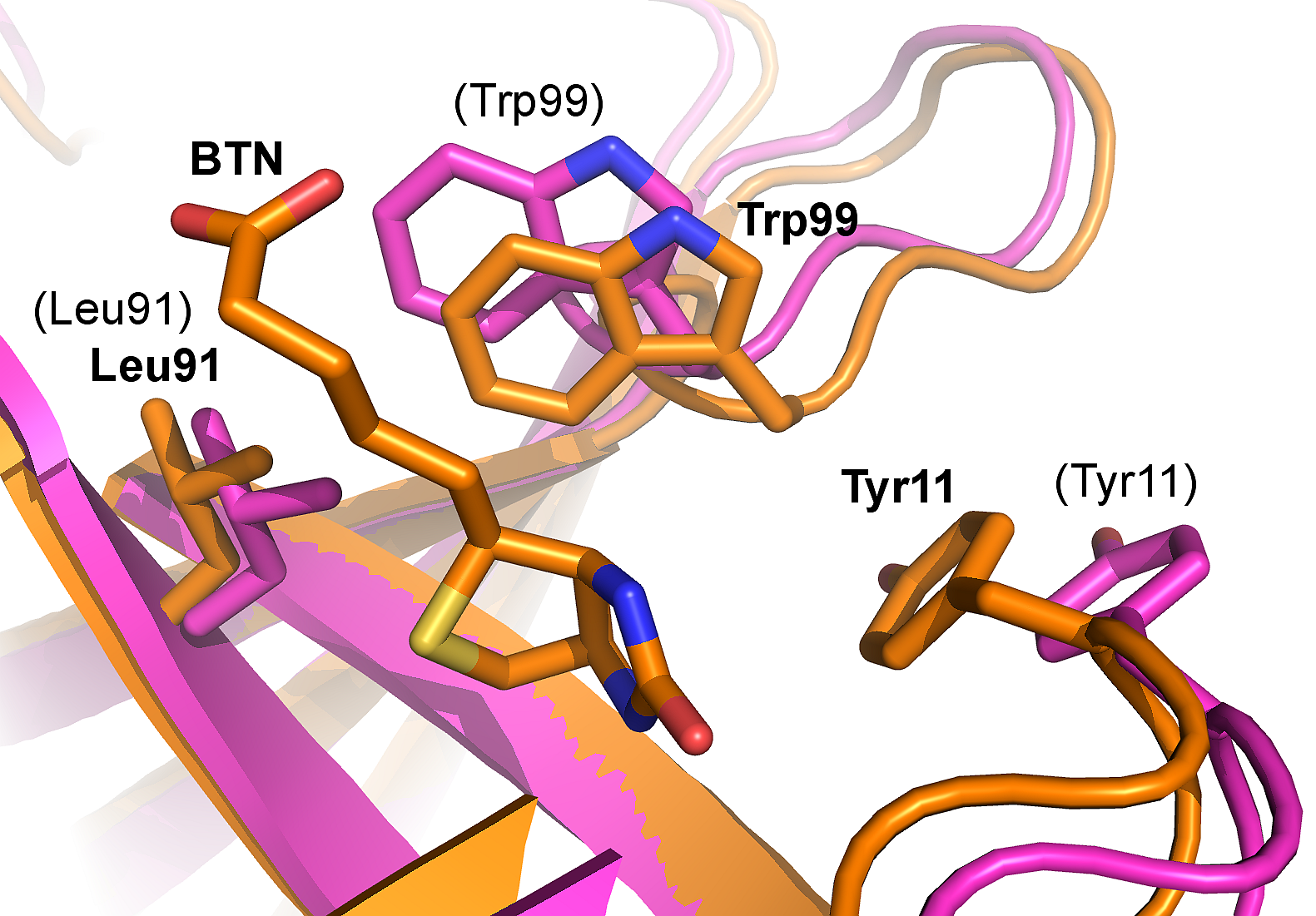

Supplement: S1 Fig — The biotin molecule (BTN) of the core-bradavidin structure and the side chains of Trp99, Leu91 and Tyr11 are shown as sticks. Nitrogen atoms are coloured blue, oxygen atoms red and sulphur atoms yellow. (TIF) [file pone.0176086.s001.tif]

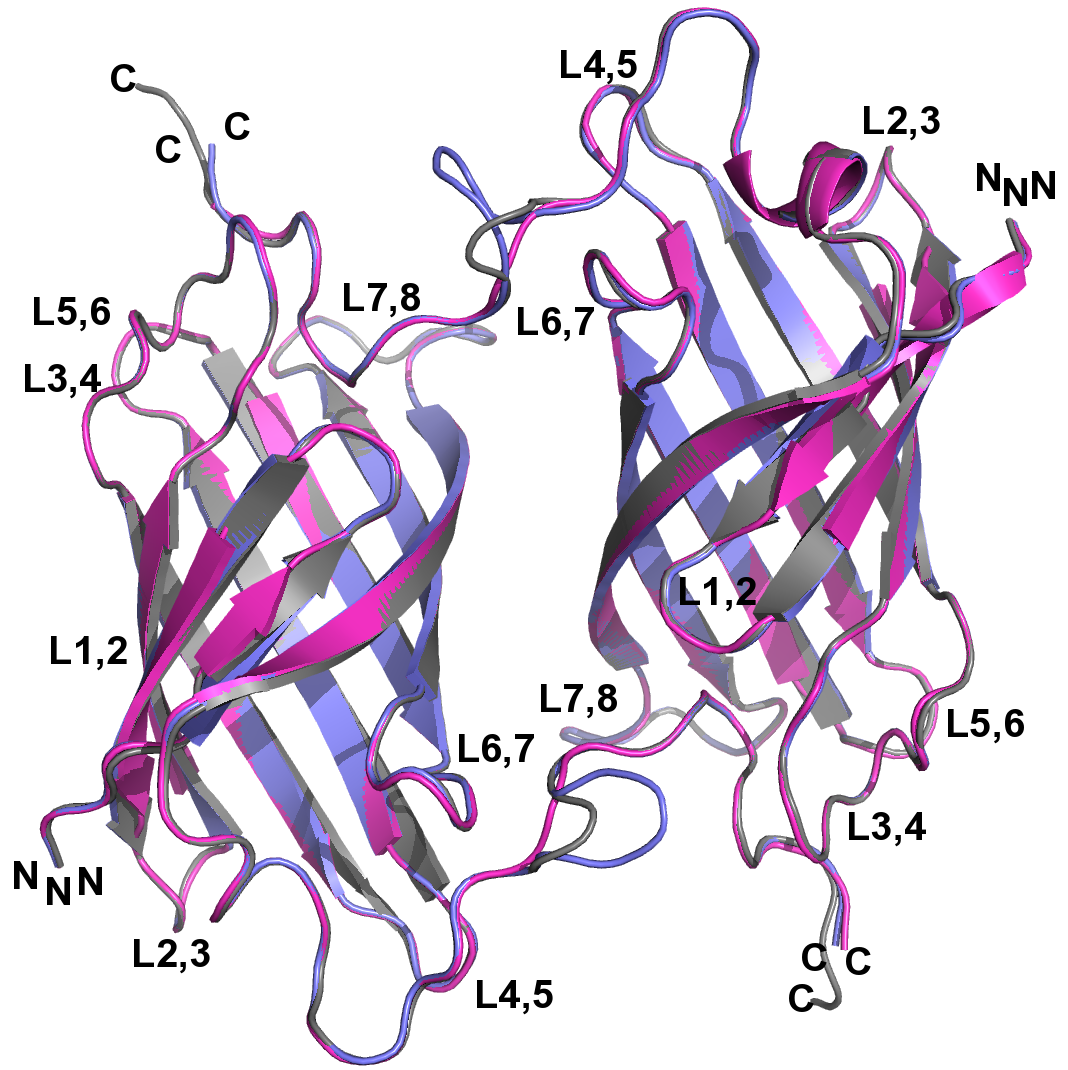

Supplement: S2 Fig — Superimposition of the Cα traces of subunit I and subunit III of wt bradavidin (magenta), rhodavidin (blue) and bradavidin A2 (grey) are shown. The loop regions L1,2 to L7,8, and the N and C termini, are labelled. (TIF) [file pone.0176086.s002.tif]

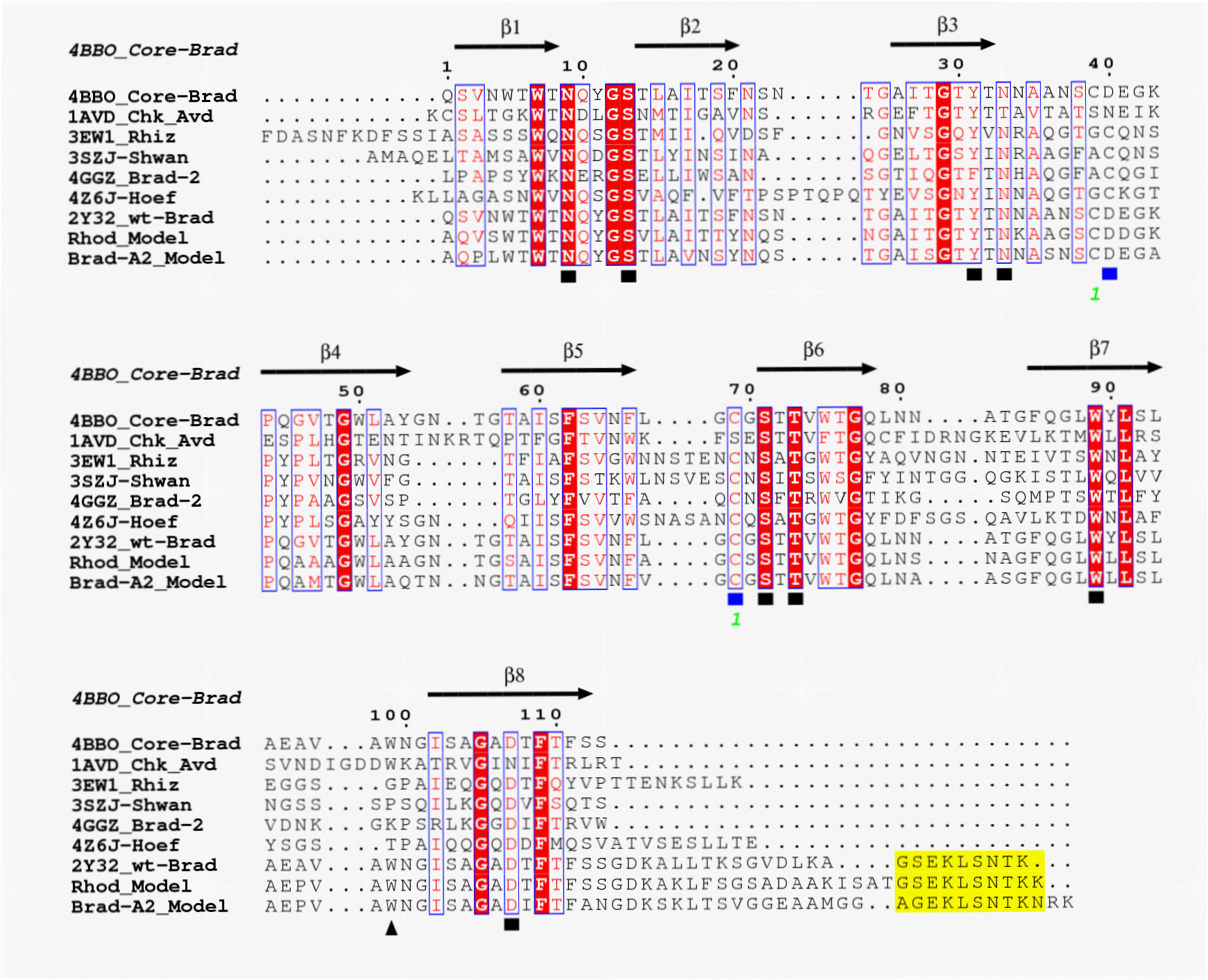

Supplement: S3 Fig — The biotin-binding residues (top six structures) are marked with black squared boxes; the blue squared boxes indicate cysteine residues forming disulphide bridges in non-tetrameric Avds; the green ‘1’ indicates the cysteine residues forming disulphide bridges in bradavidins and rhodavidin; the black triangle indicates the tryptophan residue in equivalent position to Trp99 of the core-bradavidin structure that is present only in tetrameric Avds; and the ‘Brad-tag’ residues are highlighted with yellow background. The beta-strands 1–8 of core-bradavidin are labeled and indicated by arrows. The conserved residues are coloured by the default scheme of the ESPript 3 program (http://espript.ibcp.fr/ESPript/ESPript/). (TIF) [file pone.0176086.s003.tif]
