# Supplementary material for: Structural characterization of core-bradavidin in complex with biotin
Source: PLoS One. 2017 Apr 20;12(4):e0176086. doi: 10.1371/journal.pone.0176086 (PMC5398887; doi:10.1371/journal.pone.0176086)
Supplement: S1 Table — (DOCX) [file pone.0176086.s004.docx]

# Supplementary Information

**Purified protein**

Core-bradavidin, core-bradavidin V1 and the core-bradavidin CC mutant were produced (Table S1) using existing expression constructs as described in (1,2) and *E. coli* (BL21-AI, Invitrogen) cultures of 400 ml. Protein purification was essentially done in a single step using 2-iminobiotin affinity chromatography, as described earlier in (2,3). Core-bradavidin was free of any additional residues or affinity tags, while core-bradavidin V1 and the CC mutant had 6xHis and the 3xFLAG tag (DYKDHDGDYKDHDIDYKDDDDK) attached directly on the N-terminus of the mature protein (starting from amino acids QSV). These tags were present on the purified core-bradavidin V1 and core-bradavidin CC mutant proteins, whereas their initial signal peptide (MRHFNGMLLAMIASTSLIGPLPAYA) was predicted to be cleaved off from the purified proteins based on SignalP prediction.

**S1 Table**: **Yields of core-bradavidin, core-bradavidin V1 and CC mutant produced in *E. coli* BL21-AI.**

| **Purified protein** | **Expression volume (ml)** | **Yield (mg)** | **Yield per litre (mg/l)** |
| --- | --- | --- | --- |
| Core-bradavidin Batch 1 | 400 | 7.6 | 19 |
| Core-bradavidin Batch 2 | 400 | 11 | 27.5 |
| Core-bradavidin V1 | 500 | 6.3 | 12.6 |
| Core-bradavidin CC mutant | 500 | 0.5 | 1 |

**Supplementary References:**

1. Hytӧnen VP, Laitinen OH, Airenne TT, Kidron H, Meltola NJ, Porkka EJ, et al. Efficient production of active chicken avidin using a bacterial signal peptide in Escherichia coli. Biochemical Journal. Portland Press Ltd; 2004;384(Pt 2):385.

2 Nordlund HR, Hytönen VP, Laitinen OH & Kulomaa MS. Novel avidin-like protein from a root nodule symbiotic bacterium, Bradyrhizobium japonicum. J Biol Chem; 2005;280:13250-5.

3. Hofmann K, Wood SW, Brinton CC, Montibeller JA, Finn FM. Iminobiotin affinity columns and their application to retrieval of streptavidin. Proceedings of the National Academy of Sciences. National Acad Sciences; 1980;77(8):4666-8.
